# Physiological Activity of Trace Element Germanium including Anticancer Properties

**DOI:** 10.3390/biomedicines11061535

**Published:** 2023-05-25

**Authors:** Leonid G. Menchikov, Anatoliy V. Popov

**Affiliations:** 1 N.D. Zelinsky Institute of Organic Chemistry, Russian Academy of Sciences, Leninsky Prosp. 47, 119991 Moscow, Russia; mlg@ioc.ac.ru; 2 Department of Radiology, University of Pennsylvania, 3620 Hamilton Walk, Anatomy Chemistry Building, Rm 317, Philadelphia, PA 19104, USA

**Keywords:** germanium compounds, Ge-132, germatrane, biological activity, cancer, Warburg effect

## Abstract

Germanium is an essential microelement, and its deficiency can result in numerous diseases, particularly oncogenic conditions. Consequently, water-soluble germanium compounds, including inorganic and coordination compounds, have attracted significant attention due to their biological activity. The review analyzes the primary research from the last decade related to the anticancer activity of germanium compounds. Furthermore, the review clarifies their actual toxicity, identifies errors and misconceptions that have contributed to the discrediting of their biological activity, and briefly suggests a putative mechanism of germanium-mediated protection from oxidative stress. Finally, the review provides clarifications on the discovery history of water-soluble organic germanium compounds, which was distorted and suppressed for a long time.

## 1. Introduction

At present, germanium is widely recognized as a vital trace element, which is particularly essential for the normal functioning of the immune system and plays a significant role in cancer prevention [1,2,3,4,5,6,7]. Germanium is ubiquitously present in mammalian organs and tissues, with the highest concentration in the thymus. Germanium normalizes many physiological functions, particularly blood characteristics including pH, glucose, minerals, cholesterol, uric acid, hemoglobin and leukocytes [8,9]. Conversely, germanium deficiency can result in numerous diseases, primarily oncogenic conditions [10]. Research has revealed that cancer patients exhibit anomalously low concentrations of germanium in their blood serum [7,11,12]. Additionally, germanium levels in cancerous tissues are significantly lower than those in adjacent healthy tissues [13].

Germanium is primarily introduced into the body through the consumption of vegetable-based foods with an average daily human dose of only 0.4–1.5 mg [14,15]. Research on the determination of this element in plant raw materials unexpectedly revealed an elevated content in plants and mushrooms that are traditionally used in ethnoscience, particularly in China [7,16,17,18]. Germanium compounds in natural sources have long been considered a therapeutic agent with anticancer, antitumor, antiviral and anti-inflammatory effects [19]. Thus, the highest germanium concentrations are contained in ginseng, saprophyte mushrooms, particularly lacquered polypore (*Ganoderma lucidum*) and chaga, as well as in garlic, aloe and echinacea [20,21,22,23,24,25]. Among these, ginseng and *Ganoderma lucidum* are widely used in complex therapies of oncological diseases [26,27,28,29,30]. Germanium compounds have been shown to normalize the oxygen respiration (i.e., oxidative phosphorylation) in cells, which can retard the growth of tumors [26,31,32,33]. Restoring cell oxygen respiration is key to treating Warburg-like cancers [33]. The stimulating effect of germanium on oxidizing enzymes such as aldehyde reductase [34] has also been established. Hence, germanium-containing drugs have long attracted the attention of researchers and medical practitioners.

The antitumor activity of inorganic germanium compounds was first detected in 1928 [35]. However, the field only began to intensively develop in the 1970s, when the first water-soluble organic germanium compounds were synthesized, gaining attention due to their wide range of biological activities. This topic has been addressed in several reviews [1,5,6,8,24,36,37,38,39,40,41,42,43,44], as well as a monograph [7].

This review specifically focuses on research conducted within the past decade, during which inorganic and coordination compounds of germanium have been incorporated into medical practices alongside water-soluble organic germanium compounds [3,45,46]. Moreover, the toxicity of germanium compounds has been the subject of much controversy and confusion, and the discovery history of stable water-soluble germanium compounds has been significantly distorted. Therefore, the initial focus of this review is to elucidate the tangle of errors, inaccuracies, and myths associated with germanium. At the end of this review, the authors propose a putative mechanism for germanium-mediated cancer treatment and prevention based on the unique chemical properties of germanium.

## 2. Historical Digression and Toxicity of Germanium Compounds

**Figure 1:**
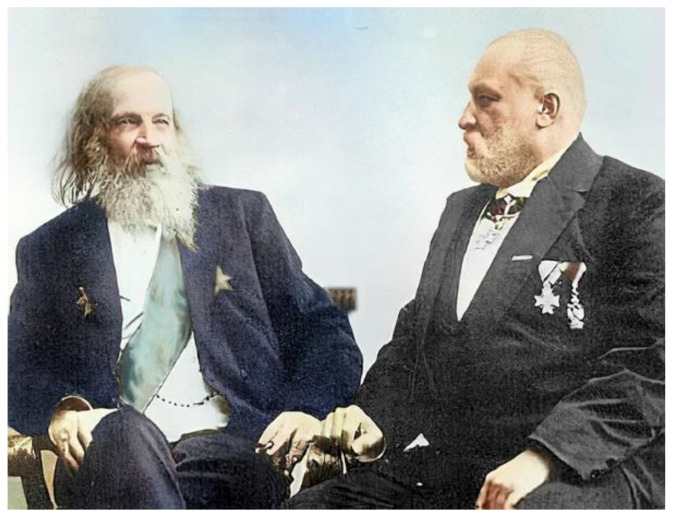
D.I. Mendeleev (**left**) and C. Winkler (**right**).

The chemical element number 32 was predicted by D.I. Mendeleev in 1871, and later, in 1886, was discovered by C. Winkler, who named it after his homeland Germany (Figure 1).

Germanium has had a tumultuous history since its discovery over half a century ago. Initially, it remained an inaccessible chemical element that did not garner much scientific attention. It was not until 1948, when the first semiconductor transistors and diodes were created using germanium, that it gained significance in the field of microelectronics. However, the use of this element as a semiconductor was soon replaced by silicon and it was again forgotten. In the 1970s, the biological activity of the discovered stable organic germanium water-soluble compounds [36] attracted the attention of scientists, among which bis(carboxyethylgermanium) sesquioxide (Ge-132) was most famous. However, in the late 1980s, interest in such compounds declined sharply as a result of an ongoing discussion about the allegedly anomalously high toxicity of organic germanium compounds (similar to organic mercury compounds). Unfortunately, the interest in such compounds declined sharply in the late 1980s due to a typo in an article published in 1987 in an inaccessible journal, which listed erroneous toxicity values for Ge-132 [6,32,33,47]. This mistake was not immediately noticed and led to erroneous criticism in subsequent publications issued in highly influential scientific journals. The correction was only published in 1988; however, until recently, many authors quoted only secondary sources that cited the erroneous data about the high toxicity of organic germanium compounds. The situation was further aggravated by a barbaric experiment conducted in Japan to determine the lethal dose of Ge-132 for humans. The experiment involved the consumption of an astronomical dose of 328 g of germanium, which is not used in medical practice [32,48,49,50]. The result of this experiment showed that the toxicity of Ge-132 was due to the formation and precipitation of solid germanium dioxide (GeO_2_) in the renal pelvis [48,49,50]. The therapeutic doses of organic germanium derivatives are thousands of times less than this lethal dose. The situation was further exacerbated by cases of germanium poisoning in individuals suffering from severe diseases, who took Ge-132 for a long time in huge excess of the recommended daily dose values without the recommendation of a doctor. These individuals consumed Ge-132 in total quantities from 15 to 300 g over a period of up to three years or more (see review [50]).

It is evident that in the instances mentioned, high doses of Ge-132 resulted in toxic effects due to its hydrolysis in the body to form solid GeO_2_ [15]. However, it is now known that such poisoning, even with extremely high doses of germanium, can be successfully treated with combined blood-purification therapy [51]. The occurrence of these tragic events led to various controversial political decisions concerning organic germanium. Specifically, Ge-132 was banned in several countries, despite being universally allowed as a dietary supplement as early as the 1980s. This resulted in the long-term neglect of research on the biological activity of Ge-132, particularly its anticancer properties. Ultimately, this denial of the role of germanium in wildlife was based on erroneous toxicity data, published in influential journals. The combination of typographical errors and reliance on secondary sources of information led to the neglect of the potential clinical use of compounds of this unique microelement. These events have also delayed the study of biological activity of germanium compounds, as noted in reviews [6,47]. To date, many influential journals continue to reject work related to the physiological activity of germanium compounds. It is now time to rectify this situation and restore justice by rehabilitating germanium and its biochemical role.

As of now, low toxicity Ge-132 has been established [40,52,53,54]. In fact, the toxicity of organic germanium compounds [55,56,57,58,59,60] is lower than that of table salt and inorganic germanium dioxide, for which the oral toxicity for mice (LD_50_) is 5400 mg/kg [55]. For example, for the best-known organic germanium sesquioxide Ge-132 oral toxicity for mice is LD_50_ > 6300 mg/kg, oral for rats is >10,000 mg/kg and intravenous toxicity for rats is >1000 mg/kg [58]. Germatranol, another common germanium derivative, is also of low toxicity: oral toxicity (LD_50_) is 8400 mg/kg for mice; intravenous toxicity is 300 mg/kg [57]. Thus, both inorganic and organic compounds of germanium are perfectly safe in those doses in which they are usually used. It should be noted that all known chemical databases, such as PubChem, currently have correct toxicity values for these compounds.

Inorganic derivatives of germanium have also been involved in a number of incidents. Dietary supplements and elixirs containing cheap both inorganic GeO_2_ and Ge (IV) coordination complexes (particularly germanium citrate and citrate-lactate) have been widely sold in Japan since the early 1970s. They were advertised primarily for cancer treatment [51], wherein the recommended daily dose of 50–100 mg was completely safe. However, a number of precedents of poisoning by such germanium compounds in persons who took such elixirs for a long time have been described. In all cases, the daily dose of germanium was arbitrarily exceeded by tens and even hundreds of times (up to 5 g GeO_2_ per day) for a long time (up to 18–24 months or more) [48,49,61,62]. As a result, the total dose of germanium in these people was between 100 and 500 g! Some of the more common symptoms of inorganic germanium poisoning include weight loss, fatigue, gastrointestinal disorders, anemia, muscle weakness and, in all cases, kidney failure [48,49,50,61,62]. Moreover, several serious fatal cases were described (see also review [50]). Because of such cases, these elixirs were banned in many countries [60]. However, in each of the above-mentioned cases of poisoning with germanium, it is necessary to understand fully and assess not only the harm from poisoning, but also the possible benefits. Patients in the last stages of cancer took these drugs (both in the form of Ge-132 and in the form of GeO_2_ and other derivative compounds) in such huge doses independently at their own risk. When taking germanium medication, even in such toxic doses, oncological sufferers, who usually live no more than 3–6 months after diagnosis, have lived 1.5–3 years or more [50,63]. Moreover, during this time, they lived a full life, contrary to the application of classical chemotherapy.

Most of these poisoning cases occurred more than 25 years ago. However, they worsened the already bad reputation of the germanium compounds. In natural compounds, germanium forms very weak chemical bonds with organic molecules, primarily with oxygen atoms. At present, there are no methods to isolate, separate and purify such substances, so the natural germanium compounds and/or its complexes have not yet been isolated and characterized. At present, scientists have drawn attention to the water-soluble synthetic germanium derivatives that make them bioavailable and enable them to be used in safe doses.

The development of water-soluble organic derivatives of germanium (i.e., containing at least one Ge-C bond) is inextricably connected with the N.D. Zelinsky Institute of Organic Chemistry of the Russian Academy of Sciences (ZIOC RAS) and its scientists. Althouge germanium sesquioxides were known long ago, they were insoluble in water. The first water-soluble derivatives were discovered in 1965 by Prof. S.P. Kolesnikov [64,65,66], at that time a graduate student in the laboratory of Prof. O.M. Nefedov [67]. These water-soluble compounds were produced by the hydrolysis of HGeCl_3_ adducts with cyclohexanone or methyl methacrylate. Later, in 1967 Prof. V.F. Mironov, a former employee of the same laboratory, similarly synthesized another stable water-soluble germanium sesquioxide-bis(carboxyethylgermanium) sesquioxide (Ge-132, CEGS), which is now the best known [68,69].

(O_1.5_GeCH_2_CH_2_COOH)*_n_*

In the 1960s, the synthesis of such compounds seemed simple only on paper and, in reality, required highly qualified chemists and specialized equipment, which was available only in a few laboratories in the USSR and the USA. However, there is often a misconception in the literature that K. Asai, a well-known popularizer and author of several books about germanium, was the first to synthesize Ge-132. In 1967, at the international scientific conference, K. Asai learned about the discovery of water-soluble germanium compounds from Soviet scientists, who later gave him samples for testing. K. Asai was the first to foresee the pharmaceutical potential of the Ge-132 [24]. The history of Ge-132 is now well-known (see e.g., [6,7,24,70,71]). It was Ge-132 that led to the active study of biological activity of germanium compounds and their application in medical practice, especially in complex cancer therapy [7,19,31,36,72]. There are clinically proven cases of the successful use of these compounds in cancer treatment; for example, the complete remission of lung cancer was achieved when taking Ge-132 [73]. The spectrum of the biological activity of Ge-132 turned out to be very extensive, with the most pronounced being antitumor activity [40,52,53,54].

Microbiological methods are another direction for the synthesis of organic germanium compounds. Thus, the yeast fermentation method produces Bio-Germanium, a medicine that acts as an effective immunostimulant, increasing the cytotoxicity of NK cells and activating immunoglobulin, B-cells and the tumor necrosis factor [19]. However, such drugs will remain outside the scope of this review.

**Figure 2:**
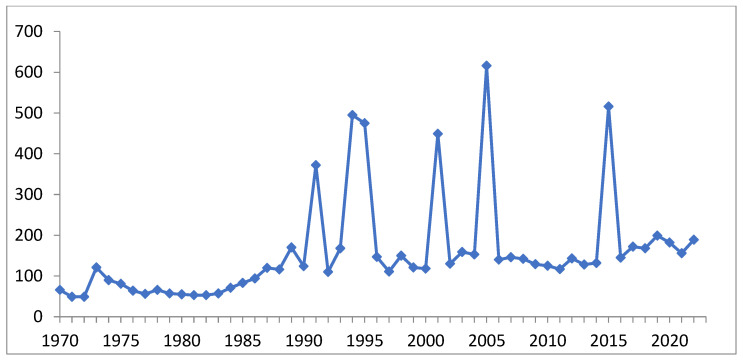
Number (per year) of publications on physiological activity of organogermanium compounds (including Ge-132) (compiled by authors of the review based on Google Academy data).

The surge in the number of publications (Figure 2) addressing the biological activity of germanium compounds until the beginning of this century was accompanied by a number of indeterminate publications containing erroneous toxicity values and reported cases of ultra-high-dose poisoning, among other issues. In the last two decades, similar peaks in publication activity were observed following the identification of novel categories of germanium compounds or the disclosure of alternative types of activity. The average number of publications has steadily risen by nearly fourfold over a span of 50 years. Thus, the discovery of novel classes of stable water-soluble germanium compounds is of significant importance.

## 3. Organic Germanium Compounds

### 3.1. Germanium Sesquioxides

**Figure 3:**
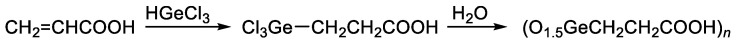
Synthesis of Ge-132.

The most-studied organic germanium compound is bis(carboxy ethylgermanium) sesquioxide (Ge-132). Its synthesis is carried out by the addition of trichlorogermane (HgeCl_3)_ to acrylic acid to produce 3-(trichlorogermyl)propanoic acid, followed by the hydrolysis thereof. In this reaction, the trichlorogermyl group Cl_3_Ge regiospecifically adds to the terminal carbon atom of the vinyl group of acrylic acids (Figure 3) [64,68,69].

Since the first synthesis of this compound was reported 55 years ago [68] the process of producing the original trichlorogermane has gone from a technically complex synthesis from elemental germanium [74] to the development of a simple and convenient method using germanium dioxide (GeO_2_), HCl and H_3_PO_2_ [75]. As a result, Ge-132 and other germanium sesquioxides are now readily available.

Regarding concerns over the alleged high toxicity of Ge-132 (see Section 2), its toxicity [56,57,58] and possible carcinogenicity [76,77] have been studied many times over the past 10 years. The results of the research once again confirmed the complete safety of Ge-132. The comprehensive study of the various biological activities of Ge-132 [8], including those that were previously known [36], particularly its antitumor [78,79,80,81,82,83,84] and immunomodulating [43,52,54,85] activities, also continued. In addition, Ge-132 is proposed as a treatment for a number of diseases: haemorrhagic and ischemic stroke [86,87], viral infections [43,54,88,89,90] (including COVID-19 [43]), various inflammatory diseases, particularly mastitis [4]. Next, this is suggested in the treatment of diabetes mellitus to reduce insulin resistance [91], and as an antioxidant for various disorders caused by oxidative stress [53,54,92,93,94] as well as in dermatological practice to heal skin wounds and protect the skin from reactive oxygen species [95,96]. Finally, Ge-132, together with hydroxyapatite, is proposed for the recovery and regeneration of mineralized tissues, particularly bone marrow [97]. The biological activity of Ge-132 is described in detail in the recently published monograph [71].

**Figure 4:**
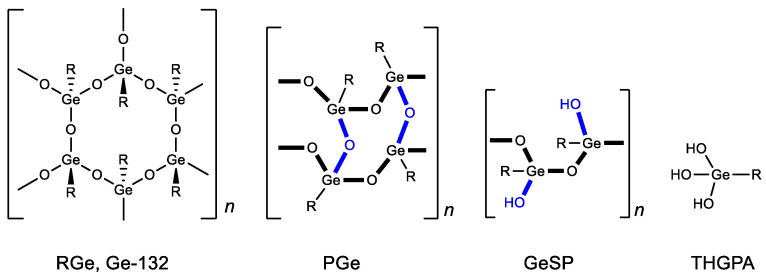
Forms of Ge-132. R = CH_2_CH_2_COOH.

Structural studies of bis(carboxy ethylgermanium) sesquioxide have shown that, in solid form, it can exist in several polymeric forms (repagermanium RGe, propagermanium PGe and linear polymer GeSP) (Figure 4) [88]. The structure of the polymer affects the rapidity and completeness of its solubility in water and, as a consequence, its biological activity and dosage. When dissolved in water, it turns into a hydrated form-3-(trihydroxygermil)propanoic acid (THGPA). PGe possesses the best water solubility.

As a result, there has recently been increased interest in Ge-132 in its most soluble form, PGe. For example, it is currently used in Japan to treat virus-hepatitis B [88]. Another direction in the study of Ge-132 biological activity is associated with the direct use of its hydrated form, THGPA. Thus, THGPA is shown to inhibit melanoma cell proliferation through phagocytosis [98]. Furthermore, it was revealed to have analgesic [99] and anti-inflammatory effects [100].

THGPA contains three hydroxy groups in its molecule, which can react with OH- groups of vital molecules. Such interactions may explain a number of physiological effects of Ge-132. Thus, to assess the possible mechanisms of this physiological activity, the interaction of THGPA with biologically active compounds such as adrenaline and ATP, which have vicinal diol functional groups, has been studied in detail. The interaction with these diols explains the numerous physiological functions of Ge-132 at low toxicities [52,100]. It was later found that, in solution, THGPA can form complexes with nucleotides or nucleosides containing *cis*-diol fragments [101]. At the same time, the ability of THGPA to form complexes with nucleotides depended on the number of phosphate groups present at the ribose residue. Interestingly, THGPA inhibits the enzymatic activity of adenosine deaminase (ADA) when using adenosine as a substrate [101].

Given the presence of several reaction centers in the Ge-132 molecule, chemical modification has been explored to increase biological activity and broaden its scope of application. Several Ge-132 derivatives have been synthesized, including those substituted on the carboxylic group, 3-alkylsubstituted, and those with substitutes on the germanium atom.

**Figure 5:**
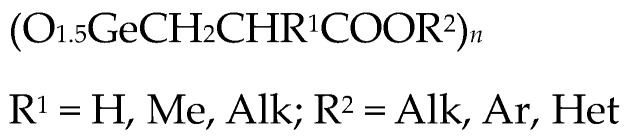
Derivatives of Ge-132.

It was previously shown that the introduction of aromatic and heteroaromatic substituents (quinolin, anthraquinone and naphthalene) as an ester group in Ge-132 increased their antitumor activity compared to Ge-132 itself [24,36]. At the same time, the introduction of an alkyl replacement in propionic acid position 2 (R^1^ = Alk) significantly reduced antitumor activity (Figure 5) [24,36].

**Figure 6:**
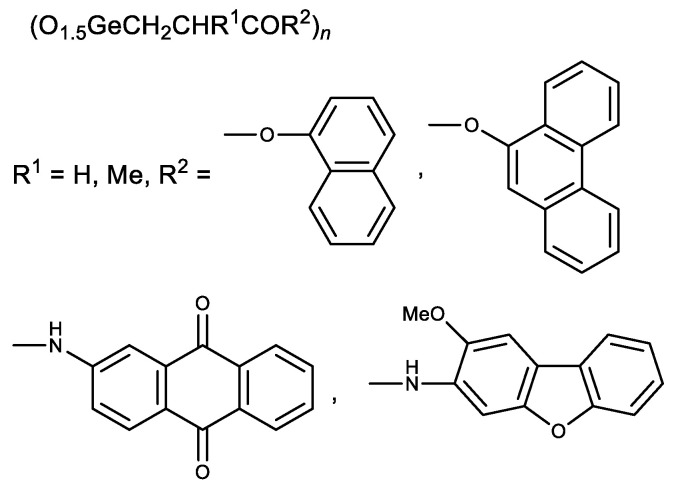
Aromatic derivatives of Ge-132.

Later esters with naphthalene and phenanthrene fragments, as well as N-arylamides with anthraquinone and dibenzofuran fragments, were synthesized (Figure 6) [102,103]. The resulting compounds had a stronger cytotoxic activity than Ge-132. The derivatives of methacrylic acid (R^1^ = Me) were therefore less active than similar derivatives of acrylic acid (R^1^ = H) [102,103]. These studies demonstrate possible means of Ge-132 modification to enhance its biological activity.

**Figure 7:**
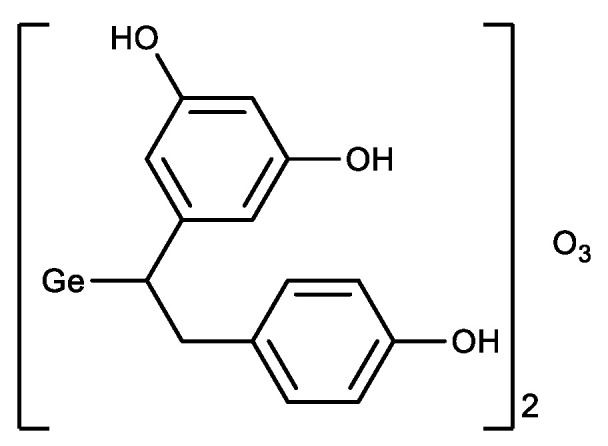
Germanium complex with resveratrol.

In parallel with the derivatives of Ge-132, a germanium sesquioxide with resveratrol was synthesized (Figure 7) [104]. The antioxidant activity of the resulting compound was higher than that of Ge-132 and resveratrol separately, i.e., a synergistic effect was observed.

### 3.2. Germatranes, Germocanes

**Figure 8:**
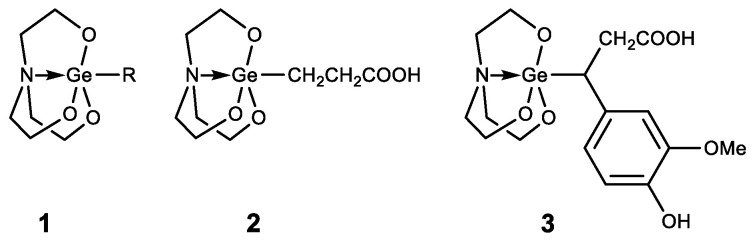
Germatranes.

Germatranes (**1**) are another interesting class of biologically active germanium compounds, which are cyclic molecules stabilized by the hypervalent germanium atom (Figure 8) [105,106,107,108,109,110].

Several compounds were identified as having high biological activity, including a peculiar hybrid of Ge-132 and germatrane-3-germatranyl substituted propionic acid (**2**) and its derivatives, which showed strong activity against various tumors [111,112,113]. Based on caffeic acid 3-germatranyl-3-(4-hydroxy-3-methoxyphenyl) propionic acid (**3**) was synthesized, which showed strong activity against cervical tumor U14 (in vitro and in vivo). This had inhibitory activity against cervical cancer cell line U14 with an IC50 as high as 48.57 mg/L (117.32 µM), whereas the degree of inhibition of the tumor growth is 64% in the animal experiment [114]. 2-aminoethoxy-substituted germatrane (**1**, R = OCH_2_CH_2_NH_2_) inhibits the activity of mononuclear alkaline phospholipase A2, and may serve for the development of new antisclerotic drugs to prevent lipid metabolism disorders [115]. In addition, this compound has a beneficial effect on the bioenergetic characteristics of mitochondria, increasing the efficiency of oxidative phosphorylation and increasing the oxidation rate of NAD-dependent substrates by mitochondria [116,117,118]. Germatranol (**1**, R = OH) reveals a similar activity; it also acts as an antioxidant and reduces the content of reactive oxygen species (ROS) in plant cells [119]. Germatranol contains a hydroxy group, which (like the hydrated form of Ge-132) can interact with functional groups in vital molecules. Thus, germatranol-hydrate interacts with simple amino acids (glycine, L-alanine, β-alanine, and L-valine), resulting in corresponding aminocarboxygermanates [120].

**Figure 9:**
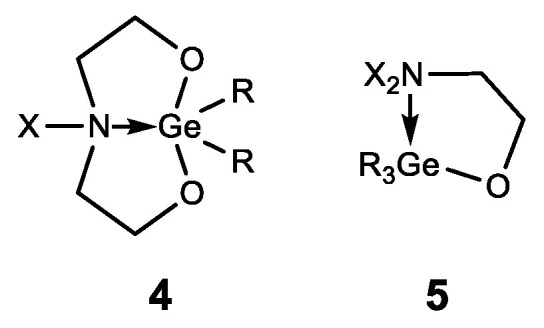
Germocanes and hypogermatranes.

In addition to germatranes, their bicyclic analogues—germocanes (quasigermatranes, **4**) and monocyclic analogues-hypogermatranes (**5**) have been synthesized, and their biological activity was found to be similar to that of germatranes (Figure 9) [108,121,122,123,124,125,126].

**Figure 10:**
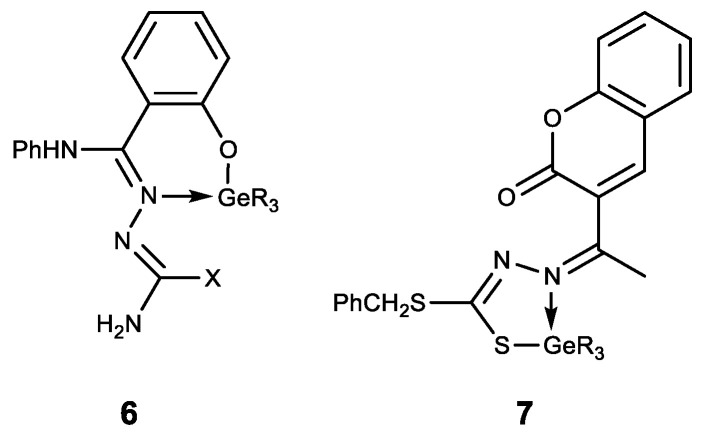
Hypogermatranes. R = Me, Ph; X = OH, SH.

The hypogermatranes **6** [127] and **7** [128] obtained in this way are molecules in which the ligands are coordinated to the germanium atom (Figure 10). These compounds exhibit antimicrobial activity against various strains of fungi and bacteria. Their pesticide activity against *Corcyra Cephalonica* is also established.

**Figure 11:**
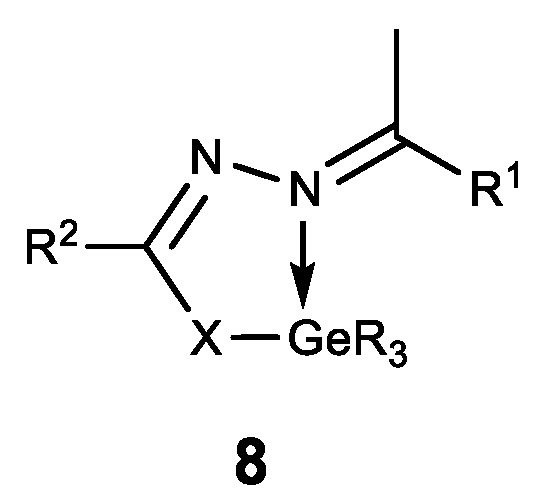
Hypogermatranes. R = Me, Ph; **8a**: R^1^ = ferrocenyl, R^2^ = NH_2_; X = O, S; **8b**: R^1^ = furan-2-yl, pyridine-2-yl, R^2^ = Py; X = O.

Hypogermatranes **8**, in which the ligands are coordinated with Ge (IV) via azomethine nitrogen atom and sulfur thiol/enol oxygen atom, are also known (Figure 11) [129,130]. These compounds have strong fungicidal and bactericidal properties. Furthermore, they are antioxidants and DNA splitters, whereas the compounds **8b** showed strong antifertile activity [130].

**Figure 12:**
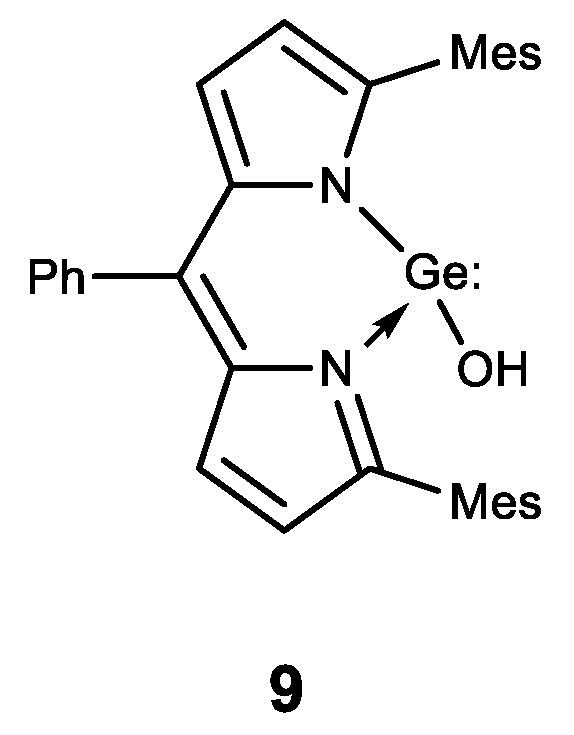
Stable water-soluble germylene.

Finally, the first stable water-soluble germylene (a compound of divalent germanium) **9** with dipyrromethane ligand was described and its biological activity was studied (Figure 12) [131]. Compound **9** has been shown to have a comparable antiproliferative effect to cisplatin. These results form the basis for further biological research using germylenes, which are highly active compounds of low-valence germanium.

### 3.3. Other Germanium Compounds

**Figure 13:**
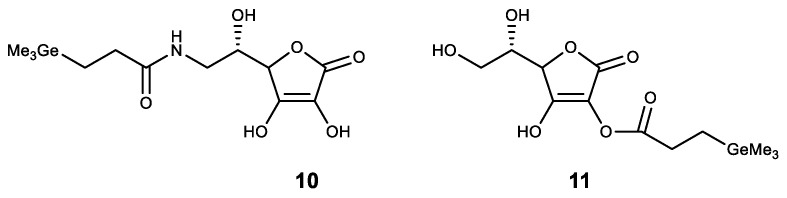
Germanium complexes with ascorbic acid.

Among the compounds of other classes, germanium was introduced to compounds with known physiological activity. The obtained compounds had a synergistic effect. One of these compounds is ascorbic acid, where germanium was introduced as a substituent. Thus, an amide of trimethylgermylpropionic acid **10** was synthesized (Figure 13). It possesses high antioxidant properties and is proposed for the treatment of atopic dermatitis [132,133]. Similarly, a stable lipophilic ascorbic acid **11** derivative with high antioxidant activity was obtained (Figure 13) [92].

**Figure 14:**
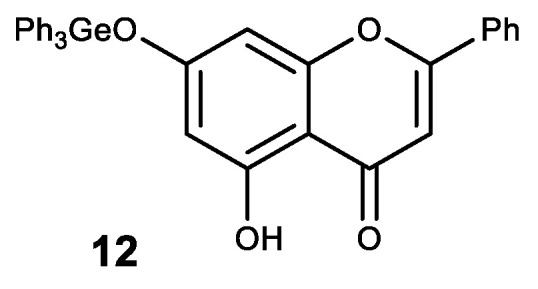
Germanium complex with crysine.

A natural flavone crysine with a wide range of biological activity was also modified in this way. The resulting germanium complex with crysine (**12**) exhibits a synergistic effect as an antioxidant (Figure 14) [134].

Complex **12** also showed high antitcancer activity. Thus, it has a significant inhibitory effect on the proliferation and growth of human cancer cell lines MCF-7, HepG2 and Colo205 with high selectivity between cancerous and normal cells [135,136]. An inhibitory effect on the proliferation of these cell lines is thought to occur through the induction of apoptosis via the ROS-dependent mitochondrial pathway [135,136].

**Figure 15:**
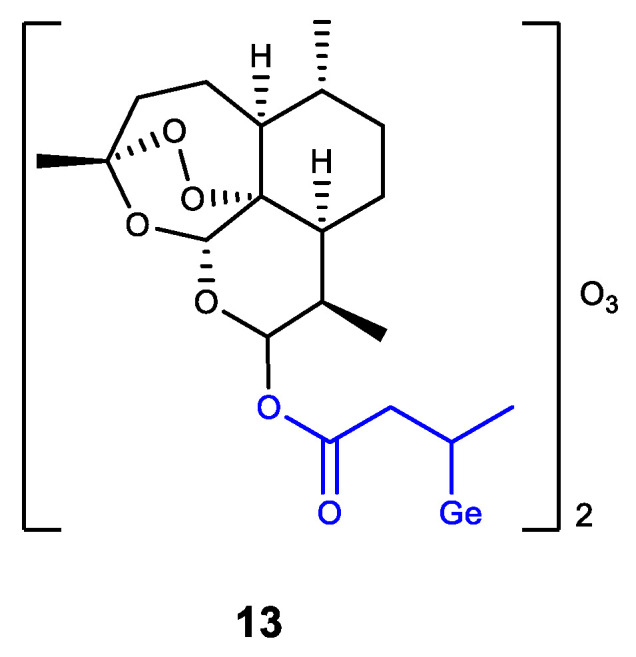
Germanium complex with dihydroartemisinin.

Germanium was also introduced into dihydroartemisinin (DHA) as an analogue of Ge-132 (product of GeHCl_3_ addition to crotonic acid) (Figure 15) [137]. The resulting DHA-Ge complex **13** displays a synergistic effect of DHA and Ge-132, i.e., effectively inhibits the proliferation of HepG2 cells and can induce their apoptosis. Complex **13** is regarded as a promising antitumor agent [137].

Steroids are another class of physiologically active compounds in which germanium was introduced as a substituent to position 16 [138,139,140]. The predicted biological activity of these and a number of other similar compounds was calculated by QSAR [141]. Antitumor, antiseborrheic and dermatological activities are the most characteristic predicted biological properties for these steroids.

**Figure 16:**
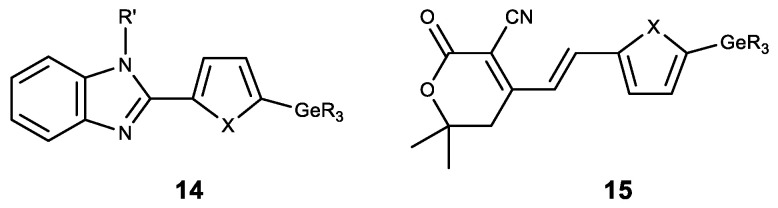
Germanium complexes with heterocycles. R = Me, Et; R’ = H, Me, Alk, CH_2_C≡CH; X = O, S.

Apart from the modification of natural compounds, GeR_3_ moeity is introduced to various heterocyclic derivatives. Thus, a number of germylsubstituted hetarylbenzimidazoles (**14**) was synthesized, and showed high cytotoxicity on the cell lines MG-22A, HT-1080 and NIH 3T3 (Figure 16) [142]. A similar series of germylsubstituted pyrane-3-carbonitriles (**15**) also showed high cytotoxicity and the inhibition of matrix metalloproteinase (Figure 16) [143]. The introduction of a germyl substituent in the heterocyclic position 5 (in furan or thiophene) was demonstrated to contribute to the emergence of cytotoxicity.

## 4. Inorganic and Coordination Germanium Compounds

The inorganic and coordination germanium compounds are now well-established in medical practice (see reviews [3,144,145] and monograph [46]). The structure of such compounds is discussed in detail in the review [146]. Problems with the use of GeO_2_ in medical practice in the 1980s were related to its low solubility, which required a substantial increase in the dose. It was recently shown to be possible to synthesize highly soluble forms of GeO_2_ [147]. This opens up new avenues for its use, including in medicine. Among the coordination germanium compounds, the most studied are germanium (IV) citrate and germanium (IV) citrate-lactate, which, like GeO_2_, are of low toxicity but exhibit nephrotoxicity in high doses [6,47,58]. These compounds activate the immune system and are recommended for the treatment of a wide range of diseases, primarily oncological [3,43,46,144,145,148].

**Figure 17:**
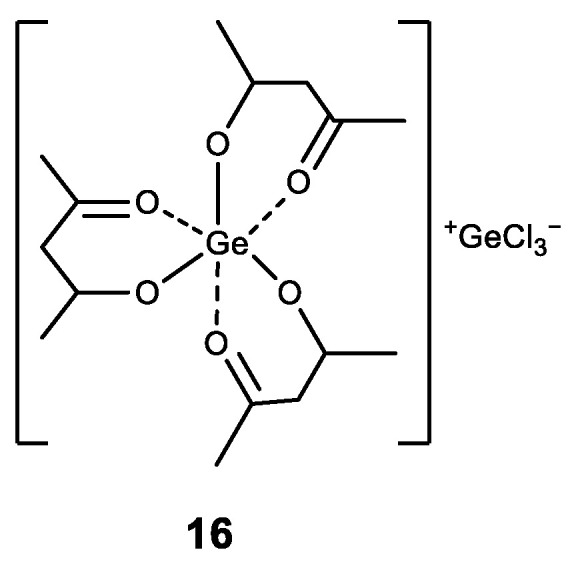
Germanium complex with acetylacetone.

There are also known complexes of germanium (IV) with acetylacetone ligand [Ge(acac)_3_)]^+^ with different anions (**16**) (Figure 17) [149]. The obtained complexes exhibit high activity against different cancer cell lines, with high selectivity in cancer cells compard to normal epithelial cells. Furthermore, the compounds induce significant apoptosis [149].

**Figure 18:**
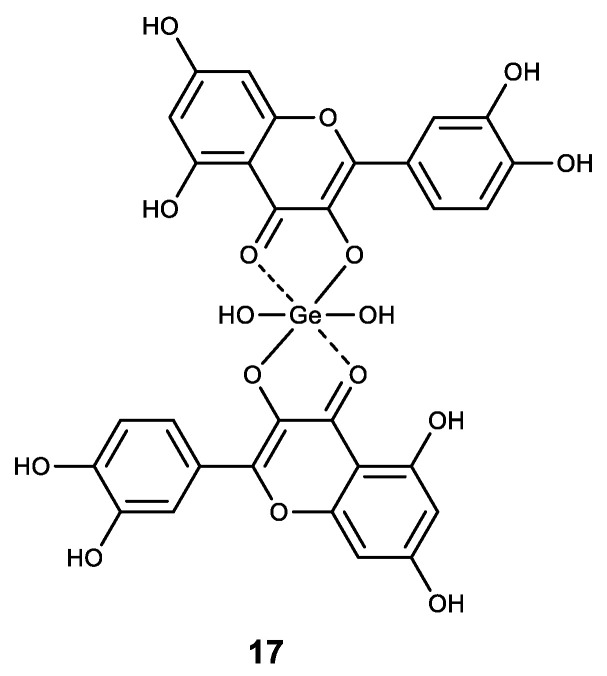
Germanium complex with quercetin.

A number of Ge (IV) complexes with natural polyphenols were synthesized and shown to be promising pharmacologically active substances for cancer treatment. The quercetin–germanium complex (**17**) (Figure 18) showed high cytotoxicity against four tumor cell lines (PC-3, Hela, EC9706 and SPC-A-1) [150,151].

**Figure 19:**
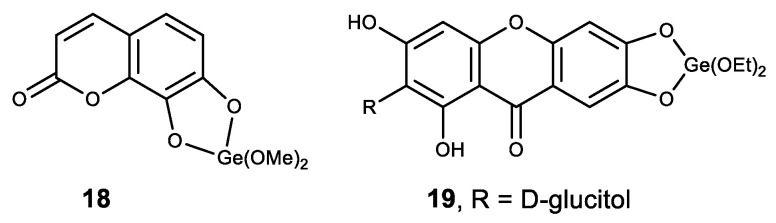
Germanium polyphenolic complexes.

Among the other polyphenolic compounds that were used in the synthesis of complexes with Ge (IV), we noted a natural coumarin daphnetin (**18**) and glucosylxanthone mangiferin (**19**) (Figure 19) [152]. The resulting Ge (IV) complexes made with the above compounds exhibit high antioxidant activity and demonstrate a strong intercalating ability in calf thymus DNA molecules. In addition, these two complexes have a strong inhibitory proliferative effect on cancer cells HepG2 [152].

Last but not least, the germanium (IV) complex with hesperidin, a flavanon glycoside, was synthesized; however the structure was not established [153]. This complex showed high activity in hepatocellular carcinoma of rats.

## 5. A Possible Mechanism of Anticancer Action of Germanium Compounds

A century ago, the Nobel Prize winner Otto Warburg observed that tumors produce excess lactate in the presence of oxygen. He proposed that the cancer’s origin lies in the replacement of oxidation phosphorylation by glucose fermentation, which he interpreted as mitochondrial dysfunction [154,155,156,157,158]. This phenomenon was called aerobic glycolysis or the “Warburg effect”. Later, the concept of mitochondrial oxidative stress was developed [159,160,161,162,163]. The mitochondrial oxidative stress leads to the over-production of ROS, which, at teh cellular level, causes aerobic glycolysis, DNA damage, autophagy/mitophagy, and protection against apoptosis [163]. During oxidative stress, the most reactive and damaging ROS is hydroxyl radical (HO^•^), which is produced from hydrogen peroxide by the Fenton reaction [164]. To protect against/prevent oxidative stress, antioxidants should be applied. Antioxidants *stoichiometrically* react with ROS. They are required in large amounts to suppress oxidative stress and can have side effects [165,166,167,168].

**Figure 20:**
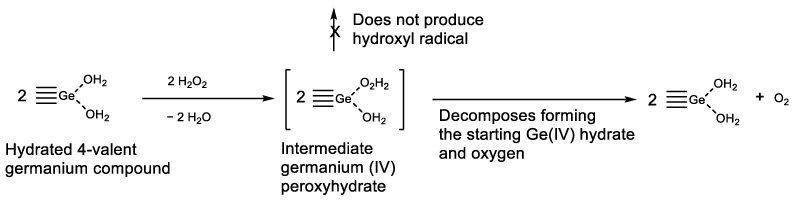
Putative mechanism of suppression/prevention of oxidative stress. Hydrogen peroxide decomposition catalyzed by Ge (IV).

Germanium compounds were found to be effective against oxidative stress [43,71,96]. Old publications describe the unique properties of germanium derivatives, which led us to suggest a putative mechanism of oxidative stress suppression/prevention. In 1930, R. Schwarz and H. Giese studied the reaction of alkali germanates with hydrogen peroxide and obtained peroxyhygrates [169]. Later, in 1935, R. Schwarz and F. Heinrich proved that these peroxyhygrates are coordination germanium compounds (not peroxides), with H_2_O and H_2_O_2_ as ligands [170]: K_2_Ge_2_O_5_·2H_2_O_2_·2H_2_O, Na_2_Ge_2_O_5_·2H_2_O_2_·2H_2_O, Na_2_GeO_3·_·2H_2_O_2_·2H_2_O. Such complexes *do not oxidize iodides and evolve oxygen*. By this means, germanium derivatives *catalytically* decompose hydrogen peroxide, and germanium trace quantities can keep hydrogen peroxide at low levels, thus dramatically reducing the formation of the HO^•^, the most damaging ROS, by the Fenton reaction (Figure 20).

Therefore, germanium derivatives can dramatically reduce hydrogen peroxide levels in cells, suppressing/preventing oxidative stress. This explains the important role of germanium in the restoration of oxygen respiration in Warburg-like cancers.

## 6. Conclusions

Germanium is a vital ultra-microelement that participates in the fundamental biochemical reactions of a living cell, determining the broadest range of biological activity in its compounds. Germanium normalizes the immune system, which is essential for cancer prevention. Germanium’s ability to restore cell oxygen respiration is particularly attractive, and can serve as the basis for the treatment of Warburg-like cancers. In addition to organic compounds, germanium’s other classes, particularly the well-known coordination compounds, have become the subject of studies of physiological activity in the last decade.

Based on the knowledge at present, it is anticipated that the exploration of biologically active germanium compounds will progress in two main directions: Firstly, through comprehensive investigations of established compounds, primarily Ge-132, aiming to obtain a more thorough understanding of their properties. Secondly, through the synthesis of novel derivatives of known compounds to enhance their biological activity and broaden their range of effects. Furthermore, research in germanium chemistry holds the potential to unveil new categories of water-soluble germanium compounds and their associated properties. Of particular relevance is the study of the mechanism of action of germanium compounds in living cells. It has been observed that germanium is integral to the active centers of certain enzymes and is involved in oxidative reactions, primarily with hydrogen peroxide, without generating detrimental reactive oxygen species, including free radicals. Consequently, germanium compounds facilitate the restoration of oxygen respiration (i.e., oxidative phosphorylation) in cancer cells, thereby impeding or even halting the growth of Warburg-like tumors. Understanding this mechanism in depth will enable the purposeful synthesis of novel germanium compounds with a targeted biological activity, yielding more significant and directed therapeutic outcomes.

Despite being neglected in a number of influential journals (see Section 2), research on the biological activity of germanium compounds continues. The reliance on secondary sources of information with erroneous data on the toxicity of organic germanium compounds is the real reason for the neglect of its biological activity to date. The publication of the review [171] has sparked further discussion on germanium, its role in wildlife, and its associated errors and misperceptions in the scientific literature [172,173].

## Data Availability

No new data were created or analyzed in this study. Data sharing is not applicable to this article.
